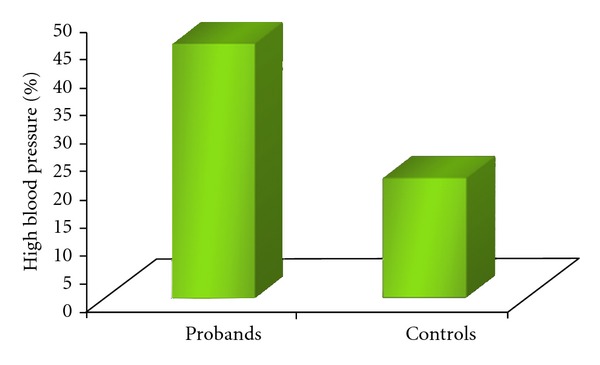# Secrecy and the Pathogenesis of Hypertension

**DOI:** 10.1155/2012/492718

**Published:** 2012-07-03

**Authors:** Randi Ettner, Frederic Ettner, Tonya White

**Affiliations:** ^1^New Health Foundation Worldwide, 1214 Lake Street, Evanston, IL 60201, USA; ^2^Clinical Instructor Feinberg School of Medicine, Northwestern University, Department of Family Medicine, 420 East Superior Street, Chicago, IL 60611, USA; ^3^Department of Child and Adolescent Psychiatry, Erasmus MC-Sophia/Kamer SP-2869, Postbus 2060, 3000 CB, Rotterdam, The Netherlands

## Abstract

Literature supporting a relationship between emotions and regulation of blood pressure dates back to the early 1900s. Theoretical explanations of the pathophysiology of the correlation have centered on several possible trajectories, the most likely being cardiovascular reactivity to stress. Prospective studies have demonstrated that chronic stress and enduring traits such as defensiveness and anxiety, impacts the development of hypertension. An analysis of 195 genetic males seeking contrary hormones for treatment of gender dysphoria revealed a significantly increased prevalence of hypertension in this cohort. The authors attribute this increased prevalence to the known effects of emotional disclosure on health and conclude that the inhibition of emotional expressiveness is significant in the etiology and maintenance of essential hypertension in this population. As hypertension is associated with morbidity and mortality, the implications for the family medicine physician treating gender nonconforming individuals and other patients in the context of a general medical practice will be discussed.

## 1. Introduction

Early psychosomatic theorists proposed that anger, hostility, and depression gave rise to hypertension [1]. Recent well-designed prospective studies document that chronic stress, as well as personality characteristics and emotional states, are associated with essential hypertension [2]. One such study evaluated 4,861 participants, aged 45–70, who lived near a major airport for at least five years. Exposure to airport noise was associated with a 14% rise in risk of hypertension [3].

Hypertension is one of the most common worldwide diseases afflicting humans. Owing to the associated morbidity and mortality, and the cost to society, hypertension is an important public health challenge. Concerted effort on the part of the health care professionals has led to decreased mortality and morbidity rates from the multiple organ damage arising from years of untreated hypertension.

Prevalence rates of hypertension vary enormously, particularly between rural and urban populations, lending credence to the importance of environmental factors in the etiology of the disease process. Populations living in rural areas typically have lower prevalence rates of hypertension than those living in urban areas. In the USA, the prevalence rate of hypertension is approximately 21–24% in individuals between 20–80 years. This rate has remained unchanged since 1999. The lowest prevalence rate worldwide is among men living in rural India, the highest prevalence rate is found in women in Poland [4].

## 2. A Proposed Pathophysiology

Chronic stress leads to an increase in adrenocorticotropic hormone (ACTH) secreted by the pituitary gland. Due to this increase in ACTH, the adrenal gland secretes excess glucocorticoids (cortisol, cortisone). Excessive cortisol production and subsequent depletion with negative sequella to the immune system, electrolytes, renal, calcium, and phosphorous bone metabolism, result in endocrine function chaos. Increased epinephrine release results in sodium retention causing elevated blood pressure, and ultimately, hypertension [5, 6]. 

Studies that examine disclosure—whether an individual shares emotionally laden personal information or keeps it secret for fear of stigmatization and shame—conclude that nondisclosure due to fear of ostracism correlates with physiological changes in tissues and organs [7]. Manuck et al. demonstrated that patients with elevated blood pressure show larger cardiovascular reactions to common stressors produced in a laboratory than normotensive controls. They suggest that lower assertiveness is a measurable deficit that corresponds to this pattern of cardiovascular activity [8]. Consistent with this finding, was that of Wirtz et al., who found strong evidence for physiological hyperactivity to stress and low social support to be significant factors [9], and Hawkley et al., who found loneliness to be predictive of elevated blood pressure [10]. 

Inhibition of expression is known to be associated with hypertension. Roter and Ewart studied 542 patient-doctor interactions to see if there were significant differences between a group of patients diagnosed with hypertension and a normotensive group. Content analysis and observer ratings revealed that physicians paid less attention to the hypertensive patients, who asked fewer questions, raised fewer concerns, and volunteered less personal information, than patients who were normotensive. The authors referred to the hypertensive patients as having patterns of self-presentation marked by an inhibition of expression [11].

Individuals who experience distress about gender, that is, are gender nonconforming, experience severe gender dysphoria or transsexualism, learn early in life to suppress or inhibit feelings, and modify behavior to avoid disapproval, shame, or ostracism. Many such individuals spend years or decades engaging in “hypermasculine” behaviors or adopting stereotypical male roles and/or avocations to avoid discovery. Some persons keep their gender-variant identity a secret even from a spouse [12]. The authors hypothesized that this group would have a higher prevalence of hypertension than a control group of individuals who had not kept such a “secret” about a socially volatile topic for a protracted period of time.

## 3. Hypothesis

In Western society, gender nonconformity requires repressing feelings and behaviors in attempts to avoid social derision, and starts at a young age. Such repression, over time, creates a scenario of chronic secrecy and inhibition of expression. Inhibition of expression is a known risk factor for hypertension. Therefore, the investigators hypothesized that collectively, the transgender cohort will have elevated rates of hypertension compared to a matched control group.

## 4. Method

### 4.1. Subjects

The subjects were 195 genetic males seeking feminizing hormone treatment at a family practice clinic in an urban setting. The mean age was 40.9 years. The ethnicity of the group was 91% Caucasian, 2% African American, 5% Hispanic, and 2% Asian.

All of the gender dysphoric males were tested for endocrine abnormalities. Hormonal ranges were consistent with natal male normal reference ranges. One patient with disorder of sexual development (previously known as intersex) was excluded from the study.

The control group consisted of 216 men who sought general medical care at the same family practice clinic with no history of gender dysphoria. The mean age of this group was 39.1 years. The ethnicity of the group was 83% Caucasian, 10% African American, 5% Hispanic, and 2% Asian.

Subjects were excluded from participation if they had pathology including: stroke, myocardial infarction, pulmonary embolism, or kidney failure. Additionally, a history of alcohol or substance abuse was a basis for exclusion.

The groups did not differ in average BMI, alcohol intake or smoking behavior. All subjects were middle to upper class in socioeconomic status. 

### 4.2. Data Collection

In this cross-sectional observational study, a retrospective chart review for evidence of hypertension was conducted. Hypertension was diagnosed by a blood pressure reading of greater than 139/89 on at least three separate office visits. The blood pressure measurement was systematically performed by the physician using the following protocol.

Patient is seated comfortably for five minutes prior to measurement: feet are on the ground and the back is supported, the arm is raised to heart level. Blood pressure is measured again at the end of the office visit.

Classifications of blood pressure measurements were based on the Seventh Report of the Joint National Committee of Prevention, Detection, Evaluation, and Treatment of High Blood Pressure (expressed in mm Hg): normal: systolic lower than 120; diastolic lower than 80, prehypertension: systolic 120–139; diastolic 80–90, stage 1: systolic 140–159; diastolic 90–99, stage 2: systolic equal to or more than 160; diastolic equal to or more than 100 [13].

## 5. Results

There were no differences in age between the two groups. The control group had greater ethnic diversity (chi-square = 11.9, df = 1, *P* = 0.0005). There were no differences in average BMI measures between the two groups. 

The control group had a rate of hypertension consistent with reports of the prevalence in the general population of the United States (21.3%). As expected, rates of hypertension were significantly associated with increasing age in both groups. 

As hypothesized, and shown in Figure 1 the probands had rates of hypertension significantly greater than the controls (45.1%) (chi-square 25.4, df = 1, *P* < 0.00001).

## 6. Discussion

Essential hypertension contributes to morbidity and mortality and has been called the “silent killer.” It is, however, the most important modifiable risk factor for coronary heart disease (the leading cause of death in North America), stroke (the third leading cause), congestive heart failure, end-stage renal disease, and peripheral vascular disease. Approximately 50 million people in the United States are affected by hypertension and approximately 20% of adults worldwide. 

In a 2009 review of 82 studies, five out of seven found a significant and positive association between measures of chronic stress and hypertension. The authors conclude that “chronic stress and particularly nonadaptive stress are likely causes of sustained elevation of blood pressure [14].” The present study suggests that there is a strong association between gender dysphoria and elevated blood pressure. Such an association does not prove causality, and further studies are warranted. 

However, this and other studies suggest that lifelong compensatory reactions to social threat may result in physiological changes involving extreme cardiovascular reactivity. Family medicine physicians will encounter patients who are reluctant to share personal information, or who do not view such information as relevant to their health status or medical care. Although the physician may take a detailed medical history and ask about sexual health and personal stressors, patients may feel uncomfortable sharing information, particularly if they fear disapproval. 

Rodriguez and Kelly asked college students to write about a personal secret while imagining an accepting confidant. A second group was to imagine a nonaccepting confidant. The “accepting confidant” group reported fewer illnesses at eight-week followup than did the nonaccepting confidant group. The authors repeated the study and concluded that “when people keep personal secrets, they often do so because they fear being ostracized. …Revealing to an accepting confidant can reduce this feeling of alienation and, as a consequence, can lead to health benefits [15].”

## 7. Conclusion

Physicians who work in primary care settings must give sufficient time and encouragement to facilitate a dialogue with patients. A supportive medical ally will focus on emotional concerns and encourage patients to ask questions. The implications of this study suggest that it is, ironically, the patient who is least likely to verbalize problems who is the most in need of the attention of the physician. Providing a safe and comfortable setting wherein patients are encouraged to discuss concerns can override the impulse to minimize symptoms and difficulties. Over the long term, this may prove as important as medication and treatment compliance in decreasing hypertension and promoting health. 

## Figures and Tables

**Figure 1:**